# PINK1 and Ataxin-2 as modifiers of growth

**DOI:** 10.18632/oncotarget.16636

**Published:** 2017-03-28

**Authors:** Nesli E. Sen, Suzana Gispert, Georg Auburger

**Affiliations:** Department of Neurology, Goethe University Medical School, Frankfurt am Main, Germany

**Keywords:** tumor suppressor, neuroblastoma regression, obesity, motor neuron disease

A recent report showed PINK1 transcript levels to be up- or down-regulated by the gain or loss of Ataxin-2 function, respectively, in human blood, in a human neural cell line and in mouse tissues [1]. These observations may have profound implications for the regulation of cell growth and may be medically exploited for the treatment of cancer and neural atrophy.

PINK1 is a mitochondrial serine threonine kinase that activates ubiquitin and the ubiquitin ligase PARKIN, triggering the autophagic elimination of dysfunctional mitochondria and of invading bacteria. PINK1 and PARKIN have an established important role for cancer, as regulators of the Warburg effect, and through their tumor suppressor action [2]. Famously, the HeLa tumor cell line with its exceptional growth carries a deletion of the PARKIN gene. Similarly, the Ataxin-2 transcriptional upregulation and its recombinant overexpression were shown to contribute to the spontaneous regression of childhood neuroblastoma tumors and to apoptosis induction in neuroblastoma cells [3].

The gain-of-function of Ataxin-2 via an expansion of its polyglutamine domain also drives neural cells into apoptosis, triggering the neurodegenerative multi-system-atrophy known as SCA2 (Spinocerebellar Ataxia type 2) and contributing to the motor neuron degenerations known as ALS and FTD (Amyotrophic Lateral Sclerosis and Frontotemporal Dementia, respectively). Conversely, the loss of Ataxin-2 triggers obesity and insulin resistance, predisposing to diabetes mellitus and hypertension [4, 5]. Importantly, a highly visible article has just demonstrated that this reduction in Ataxin-2 abundance can be exploited therapeutically to postpone the appearance of motor neuron degeneration in a TDP-43 driven mouse model of ALS, reducing its pathology, and extending its lifespan [Becker-LA et al., Nature 2017; just accepted]. Another such article has confirmed that the antisense-oligonucleotide-driven knockdown of Ataxin-2 will prevent SCA2 [Scoles-DR et al., Nature 2017; just accepted].

In the current knowledge on the hierarchy of disease proteins responsible for the neurodegenerative process in Parkinson’s disease, the recent observations [1] would now place Ataxin-2 upstream of PINK1, which is known to regulate PARKIN, and the transcriptional levels of LRRK2 are dependent on PARKIN.

Experiments in human, mouse, worms and yeast over the past 2 years have elucidated also the position of Ataxin-2 within the established cell growth pathways. They confirmed that Pbp1 as the yeast orthologue of Ataxin-2 is being controlled by AMP-kinase phosphorylation signals. Human Ataxin-2 is transcriptionally induced during starvation, and the Ataxin-2 protein relocalizes to stress granules in periods of glucose deprivation or oxidative stress [6, 7]. Downstream effects of Ataxin-2 include the repression of mTOR-dependent phosphorylation signals, but also the enhancement of PINK1-dependent phosphorylation signals [1, 6–8]. Via Ataxin-2 occurs also a regulation of the cell size, of the availability of lipid and glycogen stores as alternative fuels in times of high bioenergetic demands, and of ribosomal translation during stress periods [6, 8]. These latter global effects of Ataxin-2 are canonical functions of the mTORC1 signaling complex.

**Figure 1:**
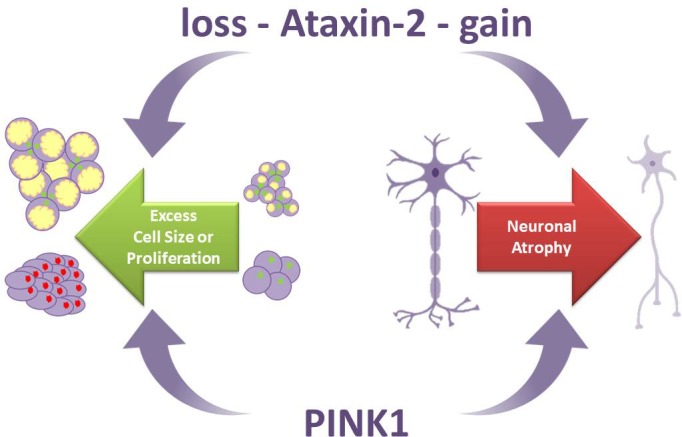
Two sides of each coin Neuronal atrophy and tumor regression are triggered by a gain of Ataxin-2 function, while Ataxin-2 deficiency is responsible for obesity and neuroprotection. PINK1 is also responsible for neurodegeneration when its function is deficient, while a gain of PINK1 function acts as tumor suppressor.

Given that the transcript levels of Ataxin-2, PINK1 and PARKIN change several fold during the transition from a nutrient excess to a starvation in amino acids, lipids and glucose [1, 6], their analysis in the blood samples or tumor tissues of patients will also provide a simple read-out that reflects trophic state versus stress responses.

Thus, Ataxin-2 represents a new target to modulate cell growth either in the direction of nutrient excess, neuroprotection, obesity, diabetes and cancer, or conversely into the direction of tumor regression, neural atrophy and apoptosis. The identification of the specific phosphorylation sites that are governing Ataxin-2 function and those phosphorylation events that depend on Ataxin-2 will be a key prerequisite to design specific drugs for the preventive treatment of a wide array of diseases.
